# Emergency laparoscopic cholecystectomy for hemorrhagic cholecystitis: A case report

**DOI:** 10.1016/j.ijscr.2021.106631

**Published:** 2021-11-27

**Authors:** Toshikatsu Nitta, Ryo Iida, Masatsugu Ishii, Masahiko Ueda, Sadakatsu Senpuku, Ayumi Matsutani, Takashi Ishibashi

**Affiliations:** Division of Surgery Gastroenterological Center, Medico Shunju Shiroyama Hospital, Osaka, Japan

**Keywords:** CT, computed tomography, HC, hemorrhagic cholecystitis, LC, laparoscopic cholecystectomy, TG18, Tokyo Guidelines, Haemorrhagic cholecystitis, Tokyo guidelines (TG18), Laparoscopic cholecystectomy

## Abstract

**Introduction:**

Hemorrhagic cholecystitis (HC) is a rare but specific complication of acute cholecystitis. HC progression often leads to fatal and severe outcomes.

**Presentation of case:**

We describe the case of a 64-year-old man who was diagnosed with HC. Intraoperatively, the gallbladder surface was congested. The resected specimen had no gallstones and showed basophilic degenerate material toward the mucosal surface. In addition, vascular congestion and red cell extravasation were noted at the bottom of the layer. The patient's postoperative course was good, and he was discharged with remission 4 days following the operation.

**Discussion:**

Diagnosis and treatment during early stages are the most critical aspects of HC management and may lead to improved outcomes.

We successfully performed timely laparoscopic cholecystectomy for the treatment of HC.

**Conclusion:**

In cases where HC occurs, appropriate treatment should be chosen by experiential judgment and consideration of the current literature.

## Highlights

• We observed that the surface of the gallbladder was congested and we diagnosed the hemorrhagic cholecystitis
• We successfully performed timely laparoscopic cholecystectomy for the treatment of hemorrhagic cholecystitis as an emergency operation.

## 1 Introduction

Hemorrhagic cholecystitis (HC) is a rare but specific complication of acute cholecystitis [1]. It may occur due to various reasons, including trauma, iatrogenic causes, malignancies, and bleeding disorders. Progression of HC often leads to fatal and severe outcomes [1].

Laparoscopic cholecystectomy (LC) has become the gold standard for managing benign biliary diseases such as stones, polyps, and cholecystitis, even in HC. LC is the treatment of choice for almost all biliary diseases because the operation is minimally invasive.

Here, we present a rare case of HC that was diagnosed preoperatively and required emergency LC.

This case has been reported in line with the SCARE 2020 criteria [2].

## 2 Case presentation

**Fig. 1:**
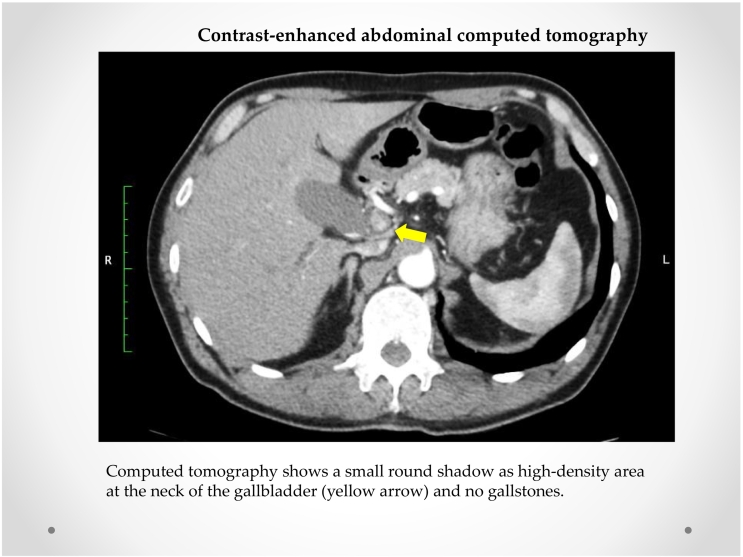
Contrast-enhanced abdominal computed tomography (CT) CT shows a small round shadow at high-density areas at the neck of the gallbladder (white arrow) and no gallstones.

**Fig. 2:**
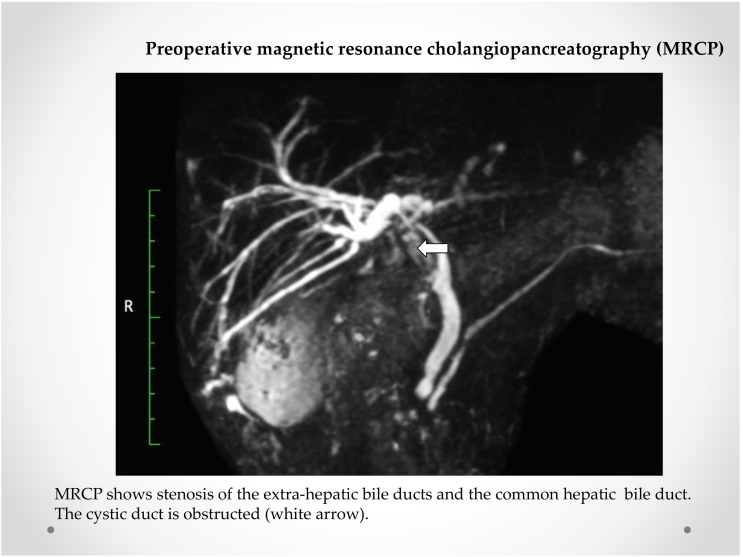
Preoperative magnetic resonance cholangiopancreatography (MRCP) MRCP shows stenosis of the extrahepatic bile ducts and common hepatic bile duct. The cystic duct is obstructed (white arrow).

A 64-year-old Japanese man presented to our hospital with right hypochondrium pain. Laboratory findings upon admission, including AST, ALT, and T-Bil levels, were near normal limits, but the hemoglobin level (11.9 g/dL) decreased (Table 1). Contrast-enhanced abdominal computed tomography (CT) detected a small round shadow as a high-density area in the neck of the gallbladder (Fig. 1); however, no gallstones were detected. Magnetic resonance cholangiopancreatography revealed compression of the extrahepatic and common hepatic bile ducts along with cystic duct obstruction. No common bile duct stones or anatomical variations of the bile duct were noted (Fig. 2).

**Table 1:** Laboratory findings.

**Peripheral blood**			**Blood chemistry**			**Serological tests**		
Variable	Range	On admission	Variable	Range	On admission	Variable	Range	On admission
WBC (/uL)	3900–9800	4900	TP (g/dL)	6.5–8.3	6.3	CRP (mg/dL)	0–0.30	0.22
RBC (/uL)	430–570	348 × 10^4^	ALB (g/dL)	3.8–5.2	3.8	HBsAg		(−)
Hb (g/dL)	13.5–17.6	11. 9	T.Bil (mg/dL)	0.2–1.2	0.5	HBsAb		(−)
Hct (%)	40.0–52.0	34.3	AST (IU/L)	10–40	21	HCVAb		(−)
Plt (/uL)	12.0–34.0	36.0 × 10^4^	ALT (IU/L)	5–45	40			
			ALP (IU/L)	106–322	204			
			γ-GTP (U/L)	12–87	232			
			LDH (IU/L)	107–230	129	**Coagulation**		
**Tumor marker**			BUN (mg/dL)	8.0–20.0	7.7	Variable	Range	On admission
Variable	Range	On admission	Cr (mg/dL)	0.61–1.04	0.66	PT (sec)	10.5–13.5	12.5
CEA (ng/mL)	0–5.0	0.7	Na (mEq/L)	135–147	141	PT (%)	70–130	82.3
CA19–9 (U/mL)	0–37	3.0	K (mEq/L)	3.3–5.0	4.1	aPTT (sec)	25–40	24.2
DUPAN-2 (U/mL)	0–150	25.0	Cl (mEq/L)	98–108	107	D-dimer (ug/mL)	1.00	1.37
SPAN-1 (U/mL)	0–30	10.4	CPK (IU/L)	45–190	110			
			ChE(U/L)	201–421	129

Based on these findings, the patient was admitted to our department of surgery at the Shiroyama Hospital. He was diagnosed with acute cholecystitis (grade II) and Mirizzi syndrome (McSherry type II) due to hemorrhagic cholecystitis (the patient was not taking anticoagulants or antiplatelets). He was scheduled for emergency laparoscopy according to the Tokyo Guidelines (TG18) [3] on the same day.

**Fig. 3:**
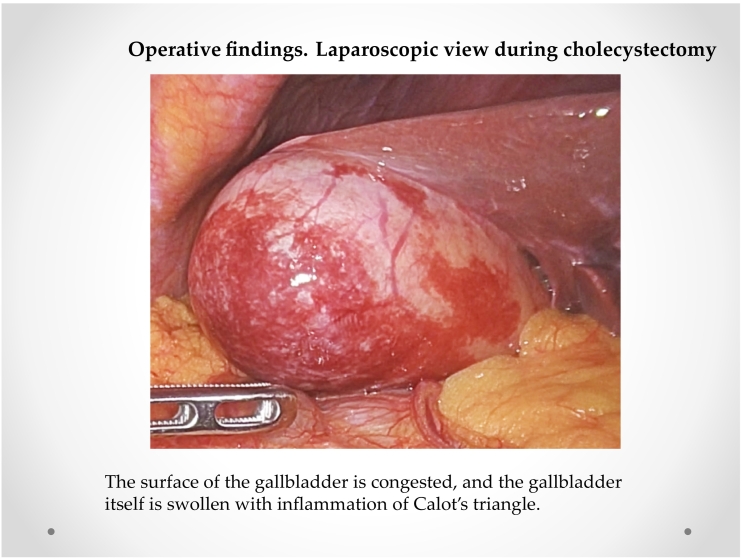
Operative findings. Laparoscopic view during cholecystectomy The surface of the gallbladder is congested and swollen with inflammation of Calot's triangle.

**Fig. 4:**
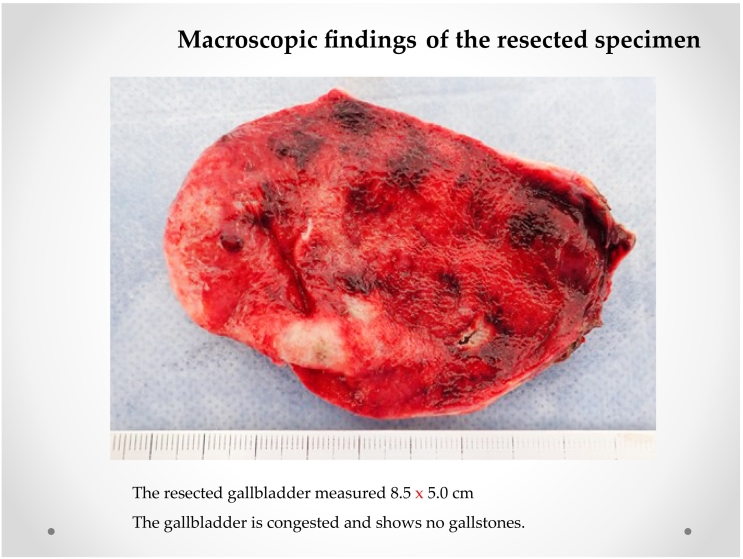
Macroscopic findings of the resected specimen The resected gallbladder measured 8.5 × 5.0 cm. The gallbladder is congested and shows no gallstones.

**Fig. 5:**
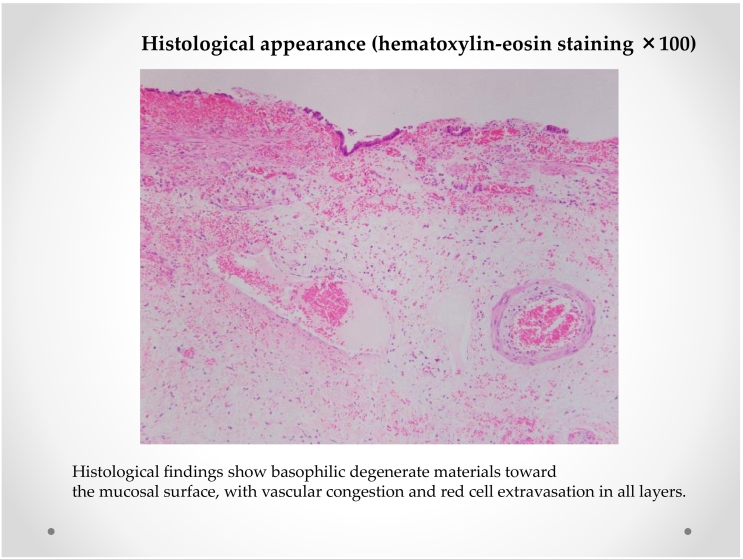
Histological appearance (hematoxylin-eosin staining ×100) Histological findings show the basophilic degenerate materials toward the mucosal surface, with vascular congestion and red cell extravasation of all layers. (For interpretation of the references to colour in this figure legend, the reader is referred to the web version of this article.)

Initially, intracorporeal procedures were performed laparoscopically using four trocars. The surface of the gallbladder was congested (Fig. 3). The gallbladder appeared swollen with inflammation of Calot's triangle, which allowed easy detection of the cystic and common bile ducts. The cystic artery and duct were skeletonized with blunt dissection, and we performed routine LC for acute cholecystitis. The resected specimen (Fig. 4) had no gallstones and showed basophilic degenerate material toward the mucosal surface. Vascular congestion and red cell extravasation were noted at the bottom of the layer (Fig. 5).

The patient's postoperative course was good, and he was discharged with remission 4 days after the operation. Written informed consent was obtained for the publication of this case report.

## 3 Discussion

Muhe first performed LC in 1985 [4]. LC became a widely accepted surgical treatment for acute and chronic cholecystitis and symptomatic cholelithiasis during the 1990s [5] and is currently the treatment of choice for such cases [6]. However, patients undergoing this procedure are occasionally admitted for emergency treatment of acute cholecystitis, including HC. Therefore, it is important to follow TG18 [3] when considering the possibility of HC. Furthermore, urgent surgical treatment, especially early LC, is recommended according to the surgeon's experience to prevent serious complications [3].

Severe HC induces shock, which is associated with a high mortality rate and requires emergency operations [7]. Therefore, diagnosis and treatment in the early stages are the most important aspects of HC management and may lead to improved outcomes. HC was first described in 1979 by Shah and Clegg [8]. HC is a part of the spectrum of cholecystitis, ranging from simple acute cholecystitis to HC, eventually progressing to gangrenous cholecystitis and gallbladder perforation [9], [10]. Fortunately, we could perform LC at an appropriate time in this case. Multiple risk factors such as anticoagulation, blunt trauma, and spontaneous hemorrhage in malignant or bleeding diathesis [11] can induce HC. In addition, gastrointestinal malignancies, including gallbladder cancer and bile tract cancer, may cause HC [8], [10], [12]. Since numerous patients are treated with various antithrombotic agents, HC should be considered when unusual presentations of cholecystitis are encountered [13].

In the present case, the patient had not received anticoagulation therapy or experienced any trauma. We could not identify the origin of the patient's HC, although severe inflammatory changes seemed to induce it. Therefore, we believe the patient's HC was a severe complication of acute cholecystitis.

In our case, (Fig. 3) were impacted, because we observed that the surface of the gallbladder was congested. The specimen showed no ulcer formation on the mucosal side of the gallbladder. Obviously, the surface of the gallbladder in this case is different from that in other cholecystitis cases. The gallbladder of HC had visibly congested vessels.

According to Nguyen et al. [14], HC is associated with calculous cholecystitis, beginning with a gallbladder obstruction, which results in increased intraluminal pressure and compromised blood flow leading to subsequent mucosal ischemia, necrosis, and erosion. Erosion of the cystic artery is much more common than that of the ensuing HC. HC has been speculated to be discordant because most of the damaged cystic arteries undergo spontaneous thrombosis, resulting in self-occlusion [15]. Occasionally, vessel wall disruption leads to the formation of a pseudoaneurysm; however, they rarely occur in patients with acute cholecystitis [16].

## 4 Conclusion

We successfully performed LC to treat HC at an appropriate time. However, surgeons performing LC should consider the possibility of HC and how it can be managed. In cases where HC occurs, appropriate treatment should be chosen by experiential judgment and after considering the literature [6].

## Sources of funding

All authors received no financial support for the research, authorship, and publication of this article.

## Guarantor

Guarantor is Takashi Ishibashi who is president of Shiroyama Hospital and my supervisor.

## Ethical approval

We got the ethical approval of this study from the ethics committee.

## Consent

We explained to the patient and relatives, and informed consent was obtained.

And we submit the certification as a guarantor.

Written informed consent was obtained from the patient for publication of this case report and accompanying images. A copy of the written consent is available for review by the Editor-in-Chief of this journal on request.

## Author contribution

We believe that this surgical report is unique and educational.

All authors engaged in the therapy of this patient.

Obviously we surgeons performed this operation as a team

This team comination of our hospital could perform these therapies.

## Registration of research studies

1. Name of the registry: Surgical strategy for Cholecystolithiasis
2. Unique identifying number or registration ID: Research Registry 5805
3. Hyperlink to your specific registration (must be publicly accessible and will be checked):

Shiroyama 2018–004

https://www.shiroyama-hsp.or.jp/patient/cancer/ethics.html

## Provenance and peer review

Not commissioned, externally peer-reviewed.

## Declaration of competing interest

All authors declared no potential conflicts of interest with the research, authorship, and publication of this article.
